# Supplementary material for: Early Time-Restricted Feeding Amends Circadian Clock Function and Improves Metabolic Health in Male and Female Nile Grass Rats
Source: Medicines (Basel). 2022 Feb 21;9(2):15. doi: 10.3390/medicines9020015 (PMC8877212; doi:10.3390/medicines9020015)
Supplement: Supplementary file 1 [file medicines-09-00015-s001.zip › medicines-1543395-supplementary.pdf]

medicines MDPI

Table S1. A list of qPCR primers was used in the study.

Genes	Forward primer	Reverse primer
Gapdh	GCCTTCCGTGTTCCTACC	CCTCAGTGTAGCCCAAGATG
Per1	CCACTGAGAGCAGCAAGAGTACA	CTGCTGCAGCCACTGGTAGA
Per2	TCACCGTAGGAGATCCGGAAT	TTTCTGCAACAGGTGCTTCCT

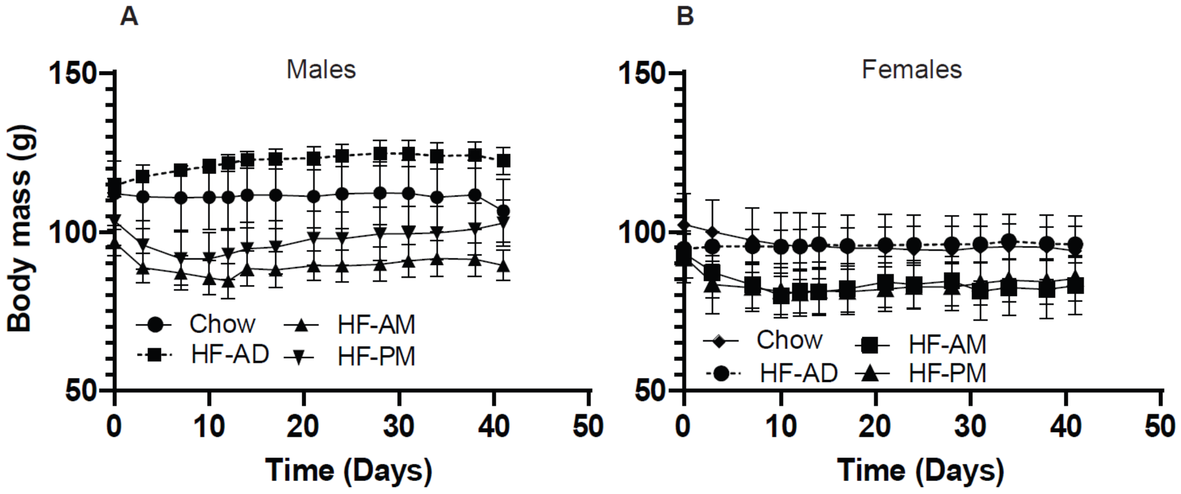

Figure S1. Time-restricted high-fat feeding of male and female NRGs. Bodyweight comparison during the intervention period between the treatment groups for male (A) and female (B) Nile grass rats. Values are mean ± SD, n= 3-5 animals/group. No significant difference was detected.

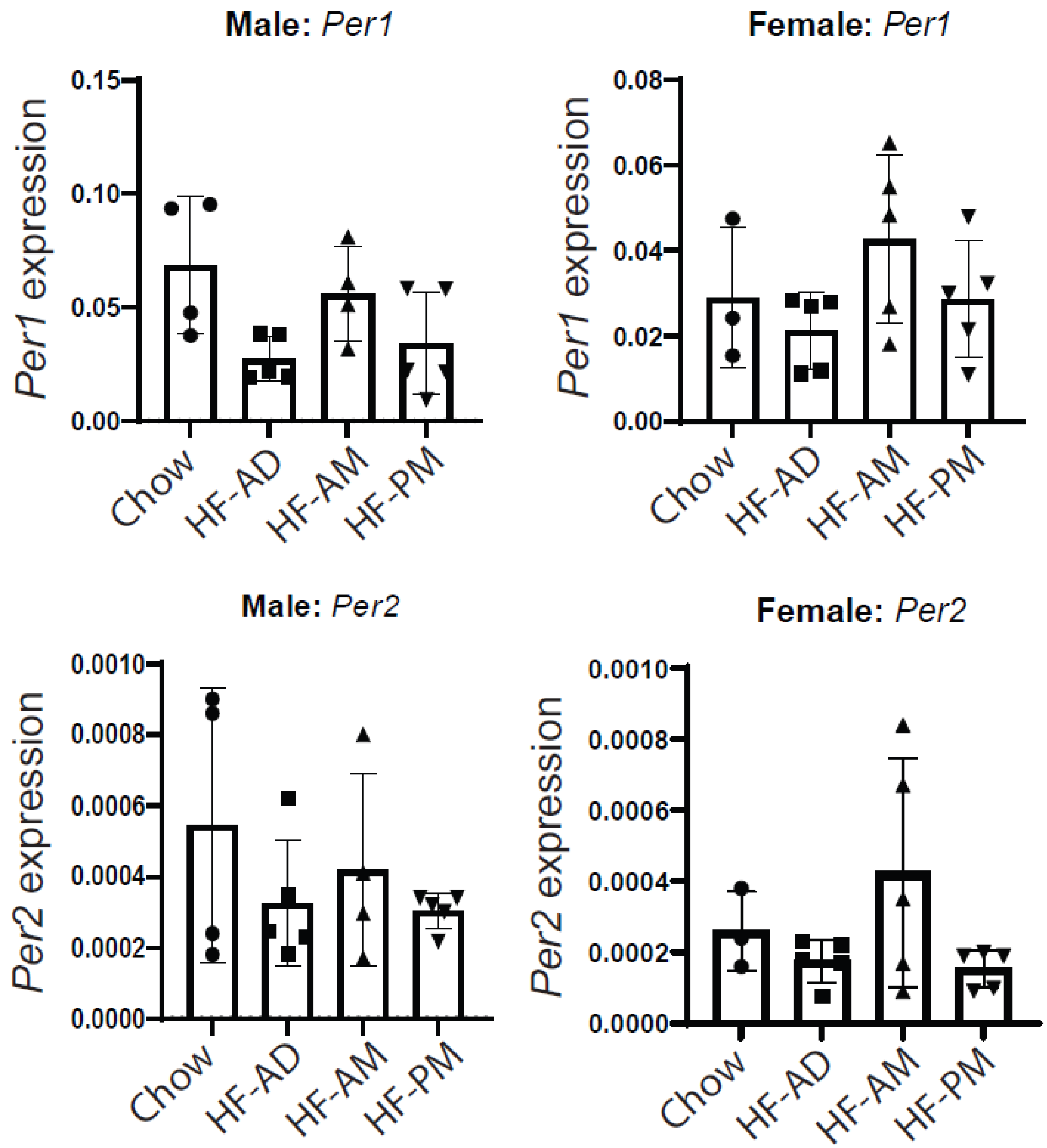

Figure S2: Clock genes Per1 and Per2 expression. Expression of Per1 and Per2 in liver samples of male and female Nile grass rats. Values are mean ± SD, n= 3-5 animals/group. No significant difference was detected.
